# Physicochemical analyses, antioxidant, antibacterial, and toxicity of propolis particles produced by stingless bee *Heterotrigona itama* found in Brunei Darussalam

**DOI:** 10.1016/j.heliyon.2019.e02476

**Published:** 2019-09-14

**Authors:** Nurul Aliah Abdullah, Fairuzeta Ja'afar, Hartini M. Yasin, Hussein Taha, Mark I.R. Petalcorin, Mitasby H. Mamit, Eny Kusrini, Anwar Usman

**Affiliations:** a Department of Chemistry, Faculty of Science, Universiti Brunei Darussalam, Jalan Tungku Link, Gadong BE1410, Brunei Darussalam; b Environmental and Life Sciences Programme, Faculty of Science, Universiti Brunei Darussalam, Jalan Tungku Link, Gadong BE1410, Brunei Darussalam; c PAPRSB Institute of Health Sciences, Universiti Brunei Darussalam, Jalan Tungku Link, Gadong BE1410, Brunei Darussalam; d Tasbee Meliponiculture Farm, Jalan Bukit Nanas, Kg Sungai Kelugos, Tutong, Brunei Darussalam; e Department of Chemical Engineering, Faculty of Engineering, Universitas Indonesia, Kampus Baru UI, Depok, 16424, Indonesia

**Keywords:** Food science, Food chemistry, Material science of foods, Analytical chemistry in food science, Chemical food analysis, Chemical characterization of food, Antimicrobial, Toxicology, *Heterotrigona itama*, Stingless bee propolis, Propolis particles, Antioxidant, Antibacteria, Toxicity

## Abstract

In this study, the physicochemical, antioxidant, antibacterial properties, and the toxicity of propolis particles produced by stingless bee *Heterotrigona itama* found in Brunei Darussalam were investigated. Propolis particles of different sizes were extracted from raw propolis using various volume fractions of ethanol in water. Spectroscopic analyses were utilized to characterize the chemical structures, functional groups, as well as absorbance and fluorescence properties. The total antioxidant capacity of propolis particles, which was assessed using DPPH (2,2-diphenyl-1-picrylhydrazyl) assay, was found to increase with volume fraction of ethanol. The maximum antioxidant capacity was as high as 317.65 mg ascorbic acid equivalent per gram of propolis particles. All of the propolis particles showed antibacterial activity against Gram-positive (*Bacillus subtilis* and *Staphylococcus aureus*) and Gram-negative bacteria (*Escherichia coli* and *Pseudomonas aeruginosa*). The diameters of the inhibition zone were either significantly higher or equivalent to those of two standard antibiotics (rifampicin and streptomycin), suggesting strong antibacterial activity. The toxicity studies of propolis particles against *Caenorhabditis elegans* revealed that they are non-toxic after 24 h exposure. Overall findings suggest that *H. itama* propolis particles are not only an important source of natural antioxidants that could be beneficial for human health, but they have potentials as antimicrobial against bacteria.

## 1 Introduction

Stingless bees, belonging to the Meliponini tribe, are eusocial bees found in tropical and subtropical regions [1]. In the ecosystem, stingless bees play an important role as pollinators for many plant species, and for production of wax, propolis, honey, and pollen [2, 3]. Propolis is a resinous adhesive (also known as bee glue), consisting of resin, beeswax, essential oils, pollen grains, micronutrients, and small amounts of vitamins [4]. In beehives, propolis is used to repair cracks and damage to the hive, as well as to defend it from predation and invasion of microorganisms [5, 6, 7]. In recent decades, a number of studies have revealed that propolis have antimicrobial [8, 9], antioxidant [10, 11, 12], antiseptic [13], anti-inflammatory [14, 15], antifungal [16], hepatoprotective and immunomodulatory properties [17, 18, 19]. The bioactivities of propolis are attributed to the presence of flavonoids, phenolic acids, terpenes, and aromatic acids, which are easily dissolved in ethanol and methanol [20].

Propolis has long been used in traditional medicines and remedies [21], and has also been utilized in food industry [22]. The biological effects and therapeutic benefits of propolis are mainly related to the synergistic effects of its chemical composition, such as phenylpropanoids, p-coumaric acid, and diterpenic acids [23, 24], which varies depending upon the stingless bee species and the plant species from which the bees collect the raw materials [7, 25]. Therefore, different combinations of chemical compounds and concentrations of propolis show diverse biological activities [26], thus, the evaluation of physicochemical properties, which can be related to the bioactivities of propolis from different geographical origins, is of importance.

As mentioned above, this study is focused on the propolis of the stingless bee species, *Heterotrigona itama*, a species found in Brunei Darussalam, locally known as *Lebah kelulut*. The genetic and behavioral characteristics of the stingless bee species have been well documented [27, 28], but studies on the physicochemical properties and the antibacterial activities of propolis from this species are scarce in the literature. The present study is therefore aimed to determine the nutritional composition and mineral contents of the propolis as well as the particle size, physical, and antioxidant properties of the propolis particles extracted using different volume fractions of ethanol in water. Considering that water is a non-toxic solvent, but it extracts less bioactive compounds when compared to ethanol, the various fractions of ethanol in water is expected to extract different biologically active compounds resulting in distinct particle size, physical, and antioxidant properties, which can be related to the bioactivity of the extracts. The *in vitro* antimicrobial activity against Gram-positive and Gram-negative bacteria and *in vivo* toxicity against *Caenorhabditis elegans* of the extracts using different ethanol-water mixtures were also investigated.

## 2 Materials and methods

### 2.1 Propolis and physicochemical analyses

The propolis of *H. itama* stingless bee species was collected from Tasbee Meliponiculture Farm in Tutong District, Brunei Darussalam. The propolis was harvested by scraping them from the bee hives during the fruiting season in early 2019. The propolis were collected 3 times (approximately 100 g each time) within two months from the same hive. It was ensured that the propolis collection did not disrupt any endangered or protected species. The physicochemical analysis, including total carbohydrates, lipids, fiber, and mineral contents of raw stingless bee propolis was determined in accordance with the phenol-sulfuric acid method, the acid hydrolysis and semi-continuous solvent extraction method, and the Weende method, respectively, as described in the official methods of analysis [29].

A wide range of elements, including Al, Ca, Cd, Co, Cr, Cu, Fe, K, Mg, Mn, Na, Ni, Pb, Zn, and As, contained in the propolis was determined using chemical reference analysis methods on inductively coupled plasma-optical emission spectrometry (ICP-OES) analyzer (iCAP 7200, Thermo Fisher Scientific) with the detection limit being 0.1 μg/kg, according to the procedure described by González-Martín et al. [30], with some modifications. The raw propolis was ground prior to analysis, and propolis powder was then digested using concentrated HNO_3_. The sample was cooled to room temperature, made up to 100 mL with distilled water, and stored at 4 °C until analysis. The calibration was performed with standard solutions in the range of 10–200 μg/kg.

### 2.2 Preparation of propolis particles

To prepare propolis nano- to micro-particles, the raw propolis was air-dried for approximately 1–2 weeks at room temperature in the dark until constant weight was obtained. Propolis extracts were prepared according to procedures reported by Jayakumar et al. [31] with modifications. Briefly, 5 g of raw propolis was cut into small pieces and suspended in 125 mL ethanol-water mixtures with different volume fractions (from 0.0 to 1.0) of ethanol (96%). The suspension was constantly agitated at 150 rpm at 37 °C in a temperature-controlled water bath for 18 h. The propolis extracts were filtered using vacuum filtration and the solvent was rotary evaporated until approximately half solvent volume reduction. The propolis extracts were then filtered through paper filter to remove the particulates, giving approximately 20 mg dried propolis particles. The extraction was repeated five times to have ample amounts of dried extracts for analysis. The collected propolis extracts were kept in colloidal suspension and stored at room temperature before further characterizations as well as antioxidant, bioactivity, and toxicity analyses.

### 2.3 Characterizations

The colloidal solutions of propolis particles in different ethanol-water mixtures were dried in oven at 40 °C. The dried extract obtained was then suspended in ultra-pure water to obtain approximately 1% w/v. To obtain a homogeneous suspension, the mixture was sonicated for 10 min. The sizes of the propolis particles extracted using different ethanol-water mixtures were measured using dynamic light scattering (Zetasizer Nano ZPS). All measurements were obtained in triplicates and the results were expressed as mean diameter ± standard deviation. Furthermore, the sizes and morphological characteristics of propolis particles were then investigated using scanning electron microscopy (SEM) (JEOL JSM-6490LA).

The vibrational spectra of propolis particles were recorded on Fourier-transform infrared (FTIR) spectrophotometer (Shimadzu Prestige-21, Japan) in the range of 400–4000 cm^−1^ with a resolution of 4 cm^−1^ using attenuated total reflection (ATR) method. The absorption spectra of the propolis particles were measured using UV-Vis spectrophotometer (Shimadzu UV-1900, Japan) in the spectral region of 200–500 nm.

The fluorescence emission of the propolis particles extracted with the various volume fractions of ethanol was analyzed using a fluorescence imaging microscope (Eclipse 50Ipol, Nikon, Japan). The samples were excited using UV-light excitation of 365 nm wavelength and the fluorescence image was captured using a CCD camera (Nikon DS-Fi1c), and the resulting images were further analyzed using ImageJ software.

### 2.4 Antioxidant assays

The antioxidant capacity of all the propolis particles extracted with different ethanol-water mixtures was determined using the 2,2-diphenyl-1-picrylhydrazyl (DPPH) free radical scavenging assay based on the procedure reported by Moreira et al. [32] with slight modifications. Briefly, the dried propolis extracts were dissolved in absolute ethanol to make a 2500 mg L^−1^ stock solution, from which various concentrations of propolis extracts were prepared. 0.5 mL of the different concentrations of propolis extracts was then mixed with 3.5 mL of ethanolic DPPH solution (50 mg L^−1^) and the mixtures were vigorously vortexed. The mixture was then left to stand at room temperature for 30 min in the dark. The decrease of DPPH radical in the mixture, as indicated by the reduction of its purple color, was quantified by measuring the absorbance of the mixture at 517 nm using a single beam UV-vis spectrophotometer (Optizen 1412V) with ethanol acting as a blank. Radical scavenging activity (RSA) of the propolis particles was determined using the following Eq. (1):

(1) $$\text{RSA}\left(\%\right)=\left(1-\frac{{\text{A}}_{\text{s}}}{{\text{A}}_{\text{0}}}\right)\times 100$$

where A_0_ and A_S_ is the absorbance of mixture without and with the propolis particles, respectively. The RSA was then plotted against the propolis concentration to give a linear plot, and the IC_50_, which was defined as the propolis concentration required to scavenge 50% initial DPPH was determined. Based on the IC_50_ value of the propolis particles extracted from different volume fractions of ethanol and that of standard ascorbic acid, the total antioxidant capacity (TAC) of the propolis particles was estimated and expressed as milligrams (mg) ascorbic acid equivalent (AAE) per gram (g) of propolis particles.

### 2.5 Antimicrobial analysis

The antibacterial activity of *H. itama* propolis particles extracted with the volume fraction of ethanol in water being 0, 0.5, and 1 were evaluated using the disc diffusion method [33] with modifications. The dried propolis extracts were dissolved in water at 20 g L^−1^. Sterile Whatman No. 1 filter paper discs (6 mm in diameter) were fully soaked with the extracts and then allowed to air dry. The bacterial screening was done on two Gram-positive bacterial strains (*Staphylococcus aureus* ATCC-29213 and *Bacillus subtilis* ATCC-11774) and two Gram-negative bacterial strains (*Escherichia coli* ATCC-11775 and *Pseudomonas aeruginosa* ATCC-27853). Each bacterial strain was cultured for 24 h at 37 °C in Nutrient Broth (Merck) and then diluted 10 fold (roughly equivalent to 0.5 McFarland standard). Petri dishes containing Mueller-Hinton agar (Bio-Rad) were each inoculated with 100 μl of the diluted bacterial culture. The dried discs containing the extracts were then placed on the agar plates. Similarly, discs containing streptomycin or rifampicin at 2 g L^−1^ were used as positive controls (reference standards) and discs containing water were used as negative control. The plates were placed in an incubator at 37 °C for 24 h before the diameter of inhibition zone as defined by the bacterial growth inhibition was measured. The results were expressed as average diameter ± standard deviation of 3–4 (propolis particles) or 2 to 3 (antibiotic) replicates. It is noted that, in this current study, the antibacterial analysis was intended to serve as a preliminary screening of *H. itama* propolis particles.

### 2.6 Toxicity studies

The assessment of toxicity was performed using synchronously grown *C. elegans* var. Bristol strain N2 (obtained from the *Caenorhabditis* Genetics Center, CGC) in 96-well plates following the method as described by Bonamigo et al. [34] with slight modifications. Briefly, 30 nematodes at L4 stage were transferred into each well in triplicates containing 50 μl of M9 buffer (3 g KH_2_PO_4_, 6 g Na_2_HPO_4_, 5 g NaCl, 0.25 g MgSO_4_ in 1 L H_2_O) as control or 50 μl of *H. itama* propolis particles at concentrations ranging from 0 to 8 mg/ml in M9 buffer.

After incubation for 24 h at 20 °C, the viability of the nematode was evaluated by assessing its movement through repeatedly touching the worms with platinum microspatula. The worm response was monitored using the Olympus SZX16 Stereo Microscope fitted with DP73 camera. The number of dead and alive nematodes was evaluated based on response. Immotile non-responsive nematodes were considered as dead while those moving were counted as alive. To prevent confusion, the dead worms were discarded while performing subsequent counting. The toxicity assessment was expressed in term of percentage mortality of the nematode.

### 2.7 Data analysis

All measurements on the raw propolis, extracted particles, and control solutions have been performed at least in triplicates and all of the data obtained was analyzed. Data were checked for normal distribution with Shapiro-Wilk test. Statistical analysis was carried out using unpaired t-test to compare significant difference between two means. A *p*-value of <0.05 was considered as statistically significant.

## 3 Results and discussion

### 3.1 Physicochemicals and minerals of *H. itama* propolis

An extensive physicochemical analysis of different nutritional parameters of *H. itama* propolis was carried out. As summarized in Table 1, the raw propolis has low compositions of crude fiber (0.30%), carbohydrates (0.43%), and protein (0.18%), but is high in lipid content (45.60%). This result can be attributed to the plant waxes and resin, which are the major components of propolis [35, 36, 37]. This also contributed to the immiscibility of the propolis and its bioactive compounds in water. The low carbohydrate content can be attributed to negligible fermentation of the propolis, as the fermentation process is linearly correlated to the carbohydrate content of substrates [38]. Proteins are the most important organic constituents and play an important role in energy production [39], while the crude fiber is thought to help with health problems, such as diabetes and high cholesterol [40].

**Table 1:** The nutritional compositions of raw *H. itama* propolis.

	*H. itama*
Crude Fiber	0.30%
Total Lipids	45.60%
Total Carbohydrates	0.43%
Crude Protein	0.18%

Table 2 summarizes the elements, including Al, Ca, Cd, Co, Cr, Cu, Fe, K, Mg, Mn, Na, Ni, Pb, Zn, and As, contained in the raw *H. itama* propolis. Amongst all of the mineral elements, K was found as the most abundant (974.24 mg Kg^−1^), followed by Mg (357.99 mg Kg^−1^) and Na (273.26 mg Kg^−1^). The metal element contents such Al, Fe, Ca, Mn, Ni, and Cu were determined as one order lower than the mineral contents. The majority of the trace toxic and heavy metal elements (Cd, Cr, Cu, Pb and As) was detected at lower concentrations, two orders lower than the mineral contents. These results agree with that reported by Gong et al. [41] on propolis obtained from China and USA with relatively high macro elemental contents with an average of more than 160 mg Kg^−1^ as well as low toxic trace elements below the detection limits.

**Table 2:** The mineral contents of raw *H. itama* propolis.

Elements	Concentration (mg Kg^−1^)	Elements	Concentration (mg Kg^−1^)
Al	33.03	Mg	357.99
Ca	18.11	Mn	34.49
Cd	0.599	Na	273.26
Co	0.438	Ni	2.20
Cr	2.74	Pb	0.75
Cu	14.58	Zn	0.24
Fe	19.84	As	4.45
K	974.24		

### 3.2 Characterizations of propolis particles

**Fig. 1:**
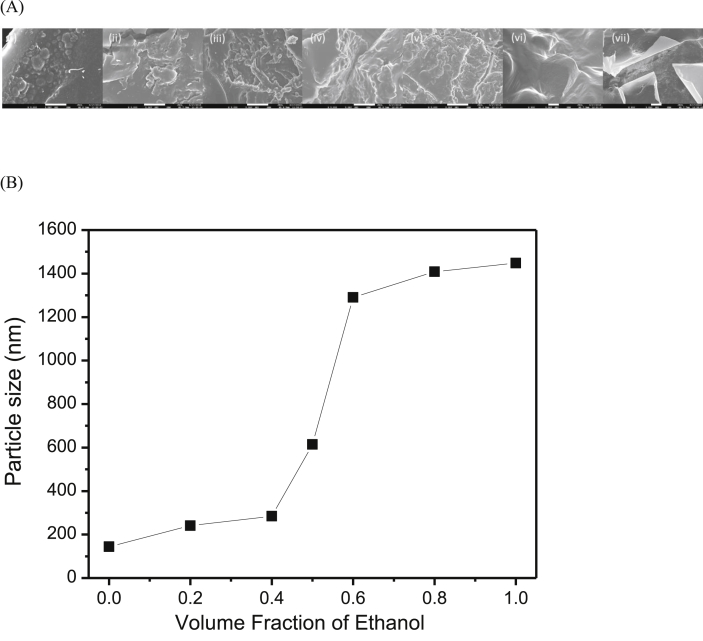
(A) SEM images of propolis particles extracted with various volume fractions of ethanol; (a) 0, (b) 0.2, (c) 0.4, (d) 0.5, (e) 0.6, (f) 0.8, and (g) 1.0; the scale bar represents 5 μm size; and (B) their respective particle sizes determined using DLS measurement.

Fig. 1A shows SEM images of the propolis particles extracted using different volume fractions of ethanol, showing the agglomerated particles which are irregular in shape with the size of a few hundred nanometers to a few microns. The sizes of the propolis particles were further confirmed by DLS measurement, as shown in Fig. 1B. The propolis particle sizes were measured to be in between 143.8 and 1448.0 nm when the volume fraction of ethanol in the mixture of solvent was changed from 0.0 and 1.0. This finding indicated that the particle size distribution increases with the volume faction of ethanol. This further implied that the addition of ethanol into water increases the amount of flavonoids, phenolics, terpenes, and aromatic acids extracted from the propolis, which can reduce the zeta potential of the particles, thereby inducing agglomeration and making larger sizes of particles. It is noteworthy that the particle sizes remain unchanged when measured after two weeks, revealing the stability of the propolis particles even in their aqueous colloidal solutions. This can be attributed to the positive surface charge on the particles, preventing their agglomeration in colloidal solutions.

**Fig. 2:**
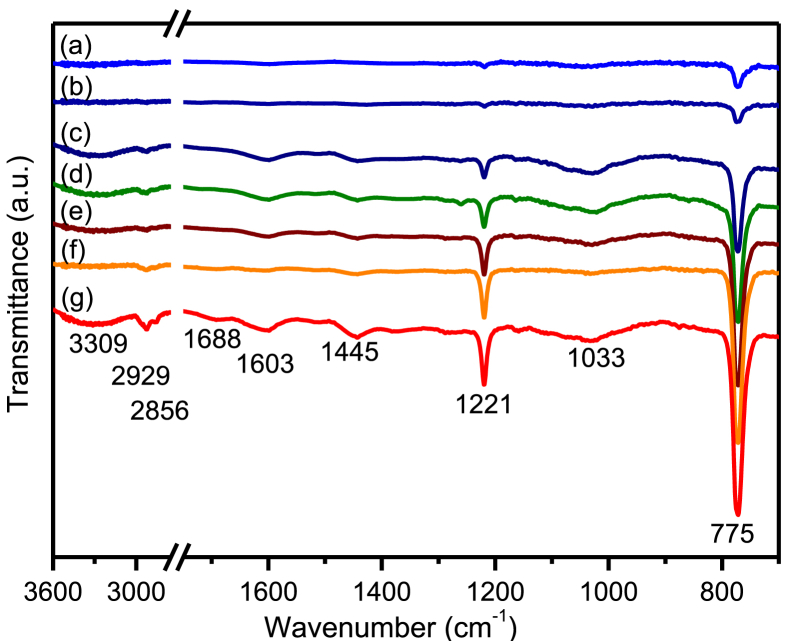
The ATR-FTIR spectra of propolis particles extracted with various volume fractions of ethanol; (a) 0, (b) 0.2, (c) 0.4, (d) 0.5, (e) 0.6, (f) 0.8, and (g) 1.0.

As shown in Fig. 2, the FTIR analysis produces consistently sharp peaks at 775 and 1221 cm^−1^ for all propolis particles extracted using different ethanol-water mixtures. This can be attributed to the out-of-plane vibrations of aromatic rings and stretching vibration of C–O bonds of polyphenols. In addition, broad vibrational bands were also observed at 1033 cm^−1^ due to the C–O stretching of ester groups and at 1445, 1603, and 1688 cm^−1^ resulting from the stretching vibrations of C–C and C–H aromatic rings and the carbonyl C=O bond. The high vibrational energy was observed for the stretching vibrations of N–H, C–H, and O–H at 2856, 2929, and 3309 cm^−1^, respectively. This finding suggests that the propolis particles contain aromatic compounds, including flavonoids, phenolic acids, terpenes, and aromatic acids [20], having mainly hydroxyl, amine, carbonyl, and ester functional groups. In Fig. 2, the FTIR analysis indicated that the vibrational peak intensities increase with the volume fraction of ethanol, suggesting that more aromatic acids were extracted in ethanol. It can be seen that the vibrational bands of the propolis particles were comparable with those of raw propolis. The phenolic compounds found in propolis as reported by different authors include, tocopherol, quercetin, vanilic, caffeic acid, ferulic acid, coumaric acid, benzoic acid, cinnamic acid, pinobanksin 5-methyl ether, apigenin, kaempferol, pinobanksin, cinnamylideneacetic acid, chrysin, pinocembrin, galangin, pinobanksin 3-acetate, phenethyl caffeate, cinnamyl caffeate, and tectochrysin [20, 34, 42, 43]. Since the propolis extraction process did not involve high temperature heating, conversion from raw propolis into nano- and micro-particles should not modify the chemical structures. Thus, one can consider that those aromatic compounds are contained in the propolis particles.

**Fig. 3:**
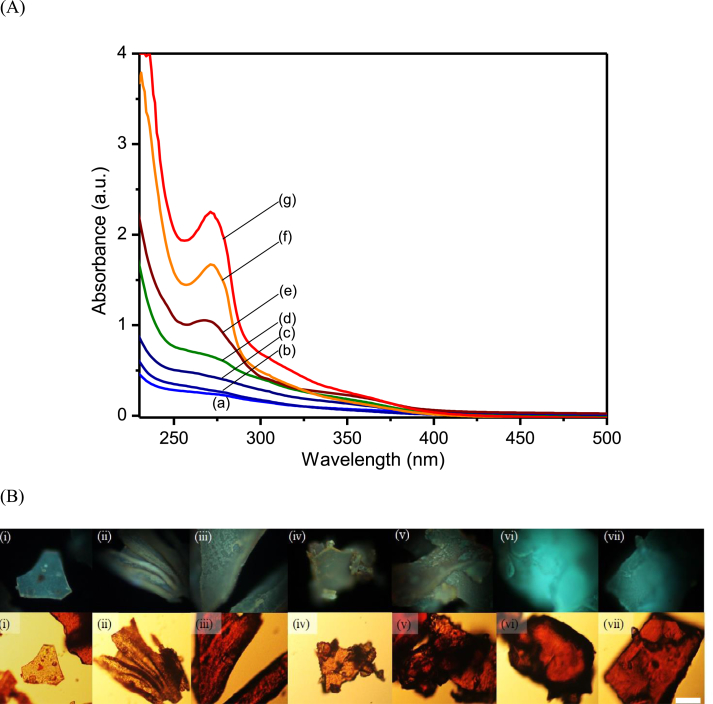
(A) The UV-Vis absorption spectrum of propolis particles extracted with various volume fractions of ethanol; (a) 0, (b) 0.2, (c) 0.4, (d) 0.5, (e) 0.6, (f) 0.8, and (g) 1.0, and (B) microscopic images (the top and bottom row is the fluorescence images and the bright-field, respectively) of respective propolis particles. The scale bar represents 30 μm size.

The UV absorption spectrum of the propolis particles showed Rayleigh scattering by the particles, in addition to an absorption band between 300 and 400 nm with a peak at 272 nm and a shoulder at 350 nm, as shown in Fig. 3A. The absorption maximum is comparable to that of propolis nanoparticles reported by Jayakumar et al. [31]. Though the absorption maximum remains unchanged, the intensity of the absorption band increases gradually with the volume fraction of ethanol, supporting the previous notion that more aromatic acids were extracted in higher volume fractions of ethanol.

This finding also suggests that the extracted aromatic compounds have chemical structures with similar π-conjugated system. In relation to the absorption spectra, fluorescence imaging of propolis particles extracted using different volume fractions of ethanol was also analyzed using fluorescence microscope with 365-nm light excitation. Fig. 3B shows the fluorescence images of propolis particles along with their respective bright-field images. It is clear that the propolis particles are fluorescent, emitting violet light, as previously reported by Jayakumar et al. [31]. While the emission color remains unchanged, the fluorescent intensity was confirmed to have a tendency to increase with volume fraction of ethanol. The increase in the fluorescent intensity demonstrates an enhancement in fluorescence quantum yield, which can be attributed to both the larger sizes and higher absorption coefficient of the propolis particles extracted with higher volume fraction of ethanol. This result suggests the potential of propolis particles to replace chemical staining for cell imaging in biomedical applications, as demonstrated by the fluorescence spectroscopic effects of Artepillin C in green propolis [31]. Here, the protonation and deprotonation of the compounds in the propolis particles could also be controlled through pH, where the change in the ionic state may influence their interactions with the cellular membrane [44].

### 3.3 Antioxidant activity

The antioxidant activities of propolis particles extracted with different volume fractions of ethanol from 0.0 to 1.0 were presented as the IC_50_ value shown in Table 3. The IC_50_ values varied from 76.5 to 1905.0 mg/L, revealing the relative amounts of antioxidants contained in the propolis particles. By comparing the IC_50_ value of the propolis particles extracted from different volume fractions of ethanol with that of ascorbic acid (24.3 ± 0.1 mg L^−1^) as the standard, TAC of the propolis particles were calculated. From the results obtained in the present work, as presented in Table 3, it is clear that the TAC of the propolis particles increase gradually with increasing volume fractions of ethanol. The highest radical scavenging activity was found in the propolis particles extracted with pure ethanol, with the TAC measured at 317.65 mg AAE g^−1^. The results supported the assumption that an increase in ethanol fraction in the extraction solvent should have higher capability to dissolve different types of phenolic compounds due to the change in the solvent polarity, leading to higher antioxidant activity expressed in the solution. These findings were in agreement with the increase in the amount of antioxidants in the propolis particles extracted at higher volume fractions of ethanol, thus unambiguously supporting FTIR and UV spectroscopic observations (Figs. 2 and 3). It is noteworthy that among the flavonoids, phenolics, terpenes, and aromatic acids contained in the propolis [20, 34], the total phenolic content has been pinpointed as responsible for the antioxidant activity of the different extracts [45, 46]. The considerable amount of total phenols in Meliponinae propolis has also been attributed to its high antioxidant capacity [47].

**Table 3:** The IC_50_ and TAC of *H. itama* propolis particles extracted with volume fraction of ethanol to water and ascorbic acid as a standard.

Volume fraction of ethanol to water	IC_50_ (mg L^−1^)	TAC (mg AAE g^−1^)
0.0	1905.0 ± 0.1	12.75
0.2	1480.3 ± 0.1	16.41
0.4	855.1 ± 0.1	28.42
0.5	669.5 ± 0.1	36.30
0.6	183.3 ± 0.1	132.57
0.8	141.3 ± 0.1	171.97
1.0	76.5 ± 0.1	317.65

### 3.4 Antibacterial activity

The propolis particles extracted using three different volume fractions of ethanol were subjected to different Gram-positive (*B. subtilis* and *S. aureus*) and Gram-negative bacteria (*E. coli* and *P. aeruginosa*). Disc diffusion assay was used to determine the concentration-dependent inhibition zones, whereby larger inhibition zone would likely indicate better antibacterial activity. All of the propolis particles were found to show antibacterial activity against the four bacterial strains. The antimicrobial activity of the propolis particles was probably due to the synergy between different phenolic compounds with various polarities, though the exact mechanism of the propolis biological activities is still unknown [48].

Table 4 shows the size of the inhibition zone in the presence of the propolis particles (20 g L^−1^) as well as two standard antibiotics, rifampicin and streptomycin (2 g L^−1^), under the same experimental conditions. For *E. coli*, the propolis particles for all volume fractions showed significantly lower inhibition zones as compared with those of each antibiotics, suggesting weaker antibacterial activity. On the other hand, for other bacterial strains, the inhibition zones were either significantly higher or statistically equivalent to the inhibition zones of the antibiotics, indicating that the propolis particles extracted in different fractions of ethanol have strong antibacterial activities against *B. subtilis*, *S. aureus* and *P. aeruginosa*. These results highlight the strong antibacterial activity of *H. itama* propolis particles against the three bacterial strains but not against *E. coli*, hence antibacterial property of the propolis is species dependent. These results are in agreement with other reported studies of stingless bee propolis, which showed high antimicrobial activities against *Campylobacter* species [49] or two different types of Gram-positive bacteria, *B. cereus* and *S. aureus* [37]. The antimicrobial effectiveness of propolis might be attributed to the synergistic activities of flavonoids, and other chemical components from the propolis particles were suggested to be responsible for structural damage to the cell wall and membrane of *Bacillus cereus*, which led to the leakage of cellular contents and hence cell death [50, 51]. Due to its strong antibacterial activity, the propolis particles have the potential for use in various biomedical applications [52, 53]. However, the underlying mechanism of the inactivation of bacterial activity still remains unclear and is deemed an interesting research prospect on future propolis development.

**Table 4:** Antibacterial activity of propolis particles extracted with three different volume fractions of ethanol to water and antibacterial activity of two antibiotics, rifampicin and streptomycin for comparison. Negative control (water) did not show any ihibition zone as expected. R means significant difference (*p* < 0.05) between the inhbition zones of the propolis particles and the respective rifampicin. Similarly, S means signifcantly different when compared to the respective streptomycin.

Bacterial strain	Zone of inhibition (mm)				
	0	0.5	1	Rifampicin	Streptomycin
*B. subtilis*	7.3 ± 1.3^S^	7.8 ± 1.7	13.0 ± 4.6	5.0 ± 1.4	4.0 ± 1.4
*S. aureus*	17.0 ± 2.5^R^	15.5 ± 2.7^R^	9.7 ± 4.6	8.5 ± 0.7	11.0 ± 4.2
*E. coli*	8.3 ± 0.5^RS^	7.5 ± 0.6^RS^	10.8 ± 1.0^RS^	18.3 ± 2.9	16.3 ± 1.5
*P. aeruginosa*	10.5 ± 1.3^RS^	12.3 ± 2.6^RS^	9.8 ± 1.3^RS^	4.0 ± 2.0	6.0 ± 2.0

Notably, the propolis particles extracted using lower volume fractions of ethanol generally showed better inhibition when compared to the commercial antibiotics, which suggests less volume fractions of ethanol for extraction was perhaps more useful for antibacterial activity. It should be noted, such comparison is usually used in many studies on the antibacterial activity of propolis. However, the comparison of the antibacterial activity of propolis extract which contains many other inactive components with the pure antibiotics is entirely qualitative, and it is only useful to screen potential natural products for antibacterial application.

### 3.5 In vivo toxicity

**Fig. 4:**
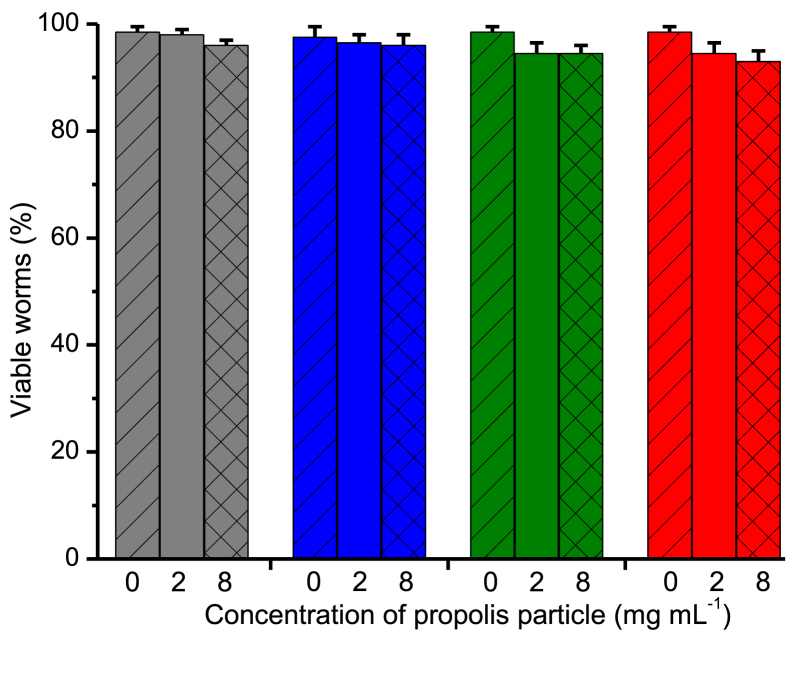
Toxicity of propolis particles at different concentrations of 0, 2 and 8 mg mL^−1^ extracted from raw *H. itama* propolis using various volume fractions of ethanol (0, 0.5, and 1.0, as presented by blue, green, and red histograms, respectively) and tested against synchronously grown L4 stage *C. elegans*. *p < 0.05 for treated versus untreated control worms. Those of raw propolis were also depicted for comparison (grey histograms).

Using whole organism *in vivo* toxicity studies, synchronously grown L4 stage worms were exposed to 0–8 mg mL^−1^ of propolis particles in M9 buffer for 24 h. The propolis particles from various extraction methods did not show any detrimental effect on the viability of the worms, as shown in Fig. 4, suggesting that propolis is not toxic to the nematodes. This confirms previous studies describing the same observation [34]. Moreover, there were reports that propolis was able to extend the lifespan and survival of worms due to its caffeic acid content that inactivates the oncogenic kinase PAK1 [54] and protects *C. elegans* against fungal infection [55].

## 4 Conclusions

In the current research work, physicochemical analyses demonstrated that raw *Heterotrigona itama* propolis contains mainly lipids (45.60%) and small amounts of carbohydrates (0.43%), fibers (0.30%), and proteins (0.18%), and it is a good source of K (974.24 mg Kg^−1^), Mg (357.99 mg Kg^−1^), and Na (273.26 mg Kg^−1^) mineral content. Extraction of raw *H. itama* propolis using various volume fractions of ethanol in water resulted in propolis particles with sizes in the range of 143.8–1448.0 nm, depending on the volume fraction of ethanol. The spectroscopic analyses, which includes vibrational, absorption, and fluorescence spectra, indicate that the propolis particles contain aromatic compounds, such as flavonoids, phenolic acids, terpenes, and aromatic acids, which mainly consists of hydroxyl, amine, carbonyl, and ester functional groups, which has an absorption at 272 nm, as well as emitting violet light fluorescence. FTIR spectra of the propolis particles consistently show peak intensity increases with increasing volume fractions of ethanol. Similarly, the total antioxidant capacity of the propolis particles, assessed using DPPH assay, also increased with volume fractions of ethanol and found to be in the range of 12.76–317.65 mg AAE per g particles. The propolis particles were demonstrated to have antimicrobial activities against different Gram-positive and Gram-negative bacteria, with the diameters of the inhibition zone observed in *S. aureus*, *B. subtilis* and *P. aeruginosa* either better or comparable with those of the two standard antibiotics (rifampicin and streptomycin) but not observed in *E. coli*. The toxicity of propolis particles against *Caenorhabditis elegans* revealed that they are non-toxic after 24 h exposure. Overall findings in study suggest that *H. itama* propolis particles are not only an important source of natural antioxidants that could be beneficial for human health, but they also have promising future prospects for antimicrobial activity against bacteria as well as a potential replacement to chemical staining agents useful in cell imaging for biomedical detection and applications.

## Declarations

### Author contribution statement

Nurul Aliah Abdullah: Performed the experiments.

Fairuzeta Ja'afar, Hartini M Yasin, Hussein Taha, Mark I.R. Petalcorin, Eny Kusrini: Analyzed and interpreted the data.

Mitasby H Mamit: Contributed reagents, materials, analysis tools or data.

Anwar Usman: Conceived and designed the experiments; Wrote the paper.

### Funding statement

This work was supported by Universiti Brunei Darussalam.

### Competing interest statement

The authors declare no conflict of interest.

### Additional information

No additional information is available for this paper.
